# Maintaining Vitality: Pharmacists’ Continuing Professional Education Decision-Making in the Upper Midwest

**DOI:** 10.3390/pharmacy6010014

**Published:** 2018-02-01

**Authors:** Paul Jacob Henkel, Marketa Marvanova

**Affiliations:** 1 Department of Geographical and Historical Studies, University of Eastern Finland, FI-80101 Joensuu, Finland; suomenpoika@yahoo.com; 2 Department of Pharmacy Practice, School of Pharmacy/College of Health Professions, North Dakota State University, Department 2650, P.O. Box 6050, Fargo, ND 58108-6050, USA

**Keywords:** community pharmacist, continuing professional education, human capital, pharmacy labor market, workforce development

## Abstract

Continuing professional education (CPE) plays an important role in continuing professional development of pharmacists for providing quality pharmaceutical care but also to maintain professional and organizational vitality and meet changing community/population needs. The study objective was to describe and understand factors of importance in selection of CPE credit hours among Upper Midwest pharmacists. A cross-sectional study of licensed pharmacists (*n* = 1239) in Iowa, Minnesota, Nebraska, North Dakota, and South Dakota included completion of a questionnaire on demographics and CPE decision-making. Factor analysis, *t*-test, and multivariate analyses were performed using Stata 10.1. Pharmacists placed greatest importance on maintaining licensure (mean = 2.72/3.00), personal interest (mean = 2.57), and self-improvement (mean = 2.42). Community/population need (mean = 1.83) was rated as slightly more important (*p* < 0.01) by retail/community pharmacists, females, and those with a Doctor of Pharmacy degree or pharmacy residency while business growth/development (mean = 1.33) was rated slightly more important (*p* < 0.01) by retail/community pharmacists. Despite findings that neither community/population need nor business development were among the most important factors in pharmacists’ CPE selection, there exists significant potential for pharmacists to utilize CPE to maintain professional and organizational vitality in the labor market, but more importantly to ensure continued provision of quality pharmaceutical care and patient education.

## 1. Introduction

Pharmacists are responsible for providing pharmaceutical care and patient education for safe and effective medication use in management of chronic and acute disease/disorders. Given expanding roles and responsibilities, pharmacists must contend with changes in population needs, pharmacy workforce issues, health care financing, and new therapeutic evidence, among other things [1]. Pharmacists must also adapt to organizational (e.g., management, budgets, health care market) and place-based changes (e.g., in- and out-migration, unemployment, living conditions) to maintain vitality as practicing health care professionals and as the pharmacy workforce. To stay current and provide safe and effective care, pharmacists must overcome such issues as: knowledge and skill atrophy, pharmacy-specific impacts of changing health care, technology advances (workplace and health care), and local firm-level or population-driven issues [2,3,4,5].

Overcoming these requires investment by professionals in the pharmacy workforce and by community pharmacies alike to contend with ongoing forces of change [6,7,8,9]. For pharmacists, from the moment that an entry-level pharmacist begins her or his first career position, the knowledge and skills gained during formal pharmacy professional education immediately become subject atrophy. In maintaining professional vitality and labor market competitiveness, pharmacists must continually seek to update knowledge and skills [10]. For pharmacies, pharmacists’ quality is important not only for quality and efficiency of pharmacy operations, but for customer satisfaction. Communication with pharmacists and perceived physical and emotional well-being are key components of satisfaction [11]. Dissatisfied customers who do or do not seek redress both engage in negative word-of-mouth (48% and 77%, respectively) [12]. Customer satisfaction and referrals are a key for maintaining competitiveness, increasingly important in the age of readily accessible online electronic reviews [12,13]. 

For many years, continuing professional education (CPE) hours have been the formal means by which the pharmacy profession has attempted to aide its constituent workforce in keeping knowledge and skills up-to-date and effectively meet population needs, and as a result remaining competitive in the labor market. CPE is not the only source of learning for pharmacists. Pharmacists learn from consuming professional literature, attending in-house trainings (e.g., grand rounds in hospitals, employer-provided training), online discussion boards, and a host of other areas, but CPE is the formal, sole requirement to maintain licensure in most states. For U.S. pharmacists, the CPE requirement varies from state to state, but is comprised of credit hours approved by the Accreditation Council for Pharmacy Education (ACPE) approved in a variety of topics and educational activities [14]. Participants in the 2015 ACPE conference on continuing pharmacy education came to the consensus that as the only formal requirement for continuing education, the current [CPE] system alone may not fully meet the needs of pharmacists in the [changing] future [15].

The aims of the study were to assess, describe and understand factors of importance in selection and CPE credit hours among registered pharmacists in the Upper Midwest. 

## 2. Materials and Methods

This cross-sectional study comprises licensed pharmacists in five U.S. states: Iowa (IA), Minnesota (MN), Nebraska (NE), North Dakota (ND), and South Dakota (SD). The study was approved by the corresponding author’s University Institutional Review Board. Pharmacists’ contact information (in-state mailing address and/or e-mail) for currently licensed pharmacists within each state were obtained from the respective state Board of Pharmacy. The survey was administered by e-mail to 100 percent of pharmacists in IA, NE, and ND, and by surface mail to pharmacists in SD. In MN, the survey was administered by surface mail to a census of pharmacists in counties (*n* = 46) with fewer than 19 pharmacists and a random sample of 19 pharmacists from each of the remaining counties (*n* = 41). Pharmacists receiving the survey by surface mail were offered the option to complete the web-based instrument, done so by 23.9% of respondents from SD and 8.6% from MN.

Eligible respondents were contacted during February to May 2017 with an invitation to participate (cover letter or e-mail body), informed about the study, that all information provided would be kept confidential and used for research purposes only, they may discontinue participation at any time, and that their voluntary completion of the questionnaire would constitute consent to participate in the study. All participants received a cover letter with study information and an electronic link to the university-hosted web-based questionnaire. Participants receiving a surface mail invitation also received a questionnaire hardcopy and a postage paid return envelope. 

The questionnaire was composed of closed- and open-ended questions. Items included primary workplace characteristics: workplace state, county of pharmacy workplace, and type (e.g., retail, hospital), and on pharmacist characteristics: terminal pharmacy degree, years of experience, residency, precepting, and membership(s) in pharmacy professional associations. Also included, were whether respondents had completed one or more Board of Pharmacy Specialties (BPS) certifications (i.e., Board Certified Ambulatory Care Pharmacist (BCACP), Board Certified Pharmacotherapy Specialist (BCPS), Board Certified Psychiatric Pharmacist (BCPP), and/or Board Certified Geriatric Pharmacist (BCGP). The questionnaire also included a battery of CPE selection criteria rated using a four-point Likert-type scale (0 = Not Important, 1 = Slightly Important, 2 = Somewhat Important, 3 = Very Important). 

Data from completed hardcopy questionnaires was entered by student assistants using a web-based entry form, downloaded as CSV files, imported into Excel, cleaned, coded, and merged with data from the web-based submissions. Statistical analyses including *t*-test and multivariate regression analyses were performed using Stata 10.1. In preliminary analysis of the CPE selection criteria data, free-of-charge and low cost for CPE where conceptually very similar and highly inter-correlated, so for each individual, these two variables were combined (free-of-charge + low cost)/2) into a single variable, No/Low Cost.

## 3. Results

There were 1239 respondents from among the five states: Iowa (IA) (*n* = 362; 29.2%), Minnesota (MN) (*n* = 209; 16.9%), Nebraska (NE) (*n* = 280; 22.6%), North Dakota (ND) (*n* = 160; 12.9%), and South Dakota (SD) (*n* = 184; 14.9%) and 44 (3.6%) valid but without state provided. Response rates varied among the five states: IA (9.7%), MN (17.3%), ND (10.9%), ND (17.8%), and SD (16.2%). Respondent pharmacists’ characteristics are described in Table 1. Respondents were likely to be female (63.3%), have completed a Pharm.D. degree (60.3%), and to have ≥10 years of pharmacy experience (70.8%). Only 13.5% of pharmacists were BPS-certified, but almost 40% served as a preceptor for a Pharm.D. Program. Pharmacists predominantly worked in institutions (40.6%) and retail/community (43.9%) settings with 2.5% in academics/research and 13.0% in other areas/specialties. Pharmacists were classified using median percent of households with at least one person aged 65 or older as: <14.0% or ≥14.0% [16]. More than 60% of pharmacists were practicing in workplaces in areas with a low population density of ≤50 persons/square mile, falling exclusively outside of metropolitan and micropolitan areas in the five states.

Maintaining licensure was the most important decision-making factor (Likert-type scale, 0–3) for all surveyed pharmacists with a mean response of 2.72 (out of 3) with the least variability from among all variables (standard deviation = 0.61). Personal interest (mean = 2.57) and self-improvement (mean = 2.42) were the second and third most important factors. Neither community/population need (mean = 1.85) nor business development/growth (mean = 1.33) were rated in the top five most important decision-making factors overall, and both ranked below no/low cost and convenience of location of CPE program. For retail/community pharmacists compared to hospital/clinic/ltc pharmacists, community/population need was significantly more important (*p* < 0.01) ranking above cost and location, and business growth was also significantly more important (*p* < 0.01) but still low in relative importance (Table 2). 

Multiple linear regression was performed to investigate the association of each CPE selection factor with workplace (community vs. institutional), population density (≤50 persons/square mile), ≥14.0% households with one person aged 65 (yes/no), completion of residency (yes/no), sex (female compared to male), terminal degree (Pharm.D. compared to B.S.), residency (yes/no), ≥10 years of experience (yes/no), BPS Certification (BCACP, BCPS, BCPP, and/or BCGP), and currently serving as a preceptor for a Pharm.D. Program (yes/no). The presented, statistically-significant, multiple linear regression coefficients for CPE selection factors (factors were standardized so beta transformation was not used) confirm that maintaining licensure, personal interest, and self-improvement were the three most important factors identified regardless of any individual, workplace, or area characteristics (Table 3). With regard to the key factors of interest, community/population need and business growth which are critical to maintain professional and organizational vitality, some interesting findings emerged. Importance of community/population need was rated higher by pharmacists working in retail/community pharmacies, females, pharmacists with a Pharm.D. and those who had completed a residency. Pharmacists in more rural areas did not place greater importance on community/population need, nor did pharmacists in areas with ≥14.0% persons aged 65 or older. Serving as a pharmacy preceptor was associated with less importance placed on community/population need or business growth as a decision-making factor for selecting CPE.

## 4. Discussion

Recent findings by Marvanova and Henkel (2017) on community pharmacists identified knowledge deficits in community pharmacists regarding Alzheimer’s disease-related knowledge, specifically inability to name adverse effects of in-stock cognitive enhancers, and inappropriate non-prescription recommendations for insomnia leading to potentially harmful drug and disease interactions [17,18]. Despite required CPE hours for licensure and efforts of the APhA and ACPE to foster lifelong learning behaviors of pharmacists, it appears CPEs are not currently effective in ensuring that pharmacists, in general, are maintaining up-to-date knowledge, at least with regard to Alzheimer’s disease [17,18]. We do not currently know if this is due to knowledge/skills atrophy, societal changes/medical advances, or both, but such deficits are detrimental to professional and organizational vitality and can negatively impact pharmacy care.

One key aspect is to understand how pharmacists in the Upper Midwest are making decisions about CPE. The study findings indicate that the most important factor guiding CPE selection is to maintain licensure followed closely by personal interest and self-improvement. Irrespective of pharmacist characteristics (i.e., years of experience, terminal pharmacy degree, completed pharmacy residency) or community characteristics (i.e., rural setting), these three decision-making factors are rated most important, in that order.

There are some factors identified that are positively associated with importance rating for selecting CPE in community/population need: working in a retail/community pharmacy setting, being female, and having completed a Pharm.D. degree or a pharmacy residency; and for selecting business growth: working in a retail/community pharmacy setting. While these were positively associated with higher importance ratings, it remains that maintaining licensure, personal interest, and self-improvement are all still considered much more important by pharmacists overall. 

The authors concern is that selecting CPE based on community/population need or its market derivation, business growth/development, are never ranked among the most important factors for selecting CPE. At best, community/population need was ranked fourth and even then only “somewhat important,” but more often than not it is rated even lower, ranking below importance of selection factors like no/low cost and/or convenience. Business growth/development is consistently rated eighth or ninth in importance, even by retail pharmacists providing pharmaceutical services in rural areas wherein there exists constant threat to organizational sustainability. To maintain vitality and competitiveness in changing labor markets in the context of continually evolving population needs and health care service provisions, prioritizing selection of CPE in community/population need and business growth/development are critical.

A statement of general consensus of a large meeting of experts in pharmacy training and development states that CPE hours are insufficient to ensure pharmacists remain current and adapt to scientific advances and changes in population and community needs and marketplace changes [15]. This, combined with growing evidence that knowledge in Alzheimer’s disease and dementia care and medications is poor despite rapidly growing aging populations [17,18], as well as with the findings of the current study, that neither community/population need nor business growth/development are among the most important selection criteria for CPE for pharmacists, does not bode well for maintaining professional or organizational vitality in a rapidly changing health care marketplace or for patient-centered care. 

A more comprehensive approach to maintaining professional vitality, lifelong learning or continuing professional development (CPD), advanced by The American Pharmacist Association (APhA) and ACPE, includes four key cyclical components of lifelong learning for pharmacists: (1) Reflection (both professional and personal) on needs; (2) Planning development in response to needs; (3) Engaging in Learning activities to meet those needs; and (4) Evaluating the effectiveness of your efforts [19,20]. Both of these leading pharmacy organizations emphasize the need for a more intentional and thoughtful approach for pharmacists’ learning post-graduating that is need-driven, systematic, and outcome-focused. 

CPD activities of pharmacists are ideally more than just CPE, though only CPE is typically required to maintain pharmacy licensure. While it is possible that non-CPE activities of pharmacists’ CPD are driven by business growth and/or local/population needs, we have no reason to suspect selection priorities for non-CPE CPD would significantly differ from CPE decision-making priorities, especially given the high level of consistency in importance identified herein, irrespective of pharmacists’ characteristics or local marketplace demographics. Moreover, there does not yet exist an effective way to monitor CPD, unlike the current requirement to select and complete annual or bi-annual CPE hours. We do strongly believe that required CPE holds significant potential for pharmacists in the contemporary and changing labor market, both as a way to maintain vitality, but more importantly, to continue to ensure provision of quality pharmaceutical care and patient education that provides for safe and effective medication use for individuals with chronic and acute disease/disorders.

Limitations of this study include cross-sectional design and inclusion of participants in only one region of the U.S. Response rates for individual states were low, between 9.7% and 17.8%. We also only investigated decision-making regarding credit-related content of CPE, and did not investigate other types of CPD. Strengths include large number of participants drawn from five states and the fact that the study findings provide evidence that confirms and elucidates what is, by general consensus, considered to be a significant problem within the pharmacy workforce and CPE practices/utility.

To further understand CPE as part of CPD and lifelong learning of pharmacists, the authors have research results forthcoming that examine CPE hours completed in the area of AD, dementia, and other chronic diseases and health issues commonly found in older adults, CPE resources, as well as research results forthcoming examining pharmacists’ utilization (or lack thereof) of a variety of formal and informal information sources on local market area/population need. Future research is planned that will investigate how pharmacists’ view changes in local organizations and populations and how they actively work to professionally adapt to perceived changes. 

## Figures and Tables

**Table 1:** Demographics of pharmacist workforce, workplace, and local market area.

Pharmacists’ (*n* = 1239) Characteristics	Percent	Percent
	Male	Female
Sex	36.7%	63.3%
	B.S.	Pharm.D.
Terminal Pharmacy Degree	39.7%	60.3%
	No Residency	Residency
Pharmacy Residency	81.1%	18.9%
	Not Certified	BPS Certified
BPS Certification ^a^	86.5%	13.5%
	Not a Preceptor	Preceptor
Pharmacy Preceptor	60.6%	39.4%
	<10 Years	≥10 Years
Years of Pharmacy Experience	29.2%	70.8%
Workplace Type/Area Characteristics		
	Hospital/Clinic/LTC	Retail/Community
Pharmacy Workplace Type ^b^	40.6%	43.9%
	<14.0% 65 and older	≥14.0% 65 and older
Households with age 65 and older	49.1%	50.9%
	≤50 persons/mi2	>50 persons/mi2
Area (county-level) population density	62.8%	37.2%

Abbreviations: Bachelor of Science in Pharmacy (B.S.); Board of Pharmacy Specialties (BPS); Doctor of Pharmacy (Pharm.D); Long-Term Care (LTC); ^a^ Board Certified Ambulatory Care Pharmacist, Board Certified Pharmacotherapy Specialist, Board Certified Psychiatric Pharmacist, and/or Board Certified Geriatric Pharmacist (formerly Certified Geriatric Pharmacist); ^b^ Retail/Community, Institutional (Hospital/Clinic/Long-term Care), Academics/Research = 2.5%, Other/Unclassified = 13.0%.

**Table 2:** Decision-making factors for pharmacist workforce (*n* = 1239) by importance.

	All Respondents		Retail/Community		Hospital/Clinic/LTC		
CPE Selection Criteria	Mean ^a^	SD	Mean ^a^	SD	Mean ^a^	SD	*T*-test ^b^
Licensure Maintenance	2.72	0.61	2.75	0.59	2.68	0.66	0.0341
Personal Interest	2.57	0.61	2.53	0.62	2.64	0.57	0.0029
Self-Improvement	2.42	0.73	2.40	0.74	2.48	0.64	0.0310
No/Low Cost	1.94	0.84	1.97	0.85	1.94	0.80	0.2549
Convenient Location	1.86	0.98	1.79	1.01	1.90	0.94	0.0376
Community/Population Need	1.85	0.92	2.02	0.87	1.79	0.87	0.0000
Employer Requirement	1.52	1.19	1.54	1.20	1.63	1.15	0.1179
Offered Online	1.43	1.06	1.33	1.07	1.54	1.03	0.0012
Business Growth	1.33	1.05	1.48	1.00	1.15	1.04	0.0000
Live Credits	0.94	1.02	0.78	0.95	1.01	1.01	0.0001
Member of Organization	0.89	0.99	0.79	0.95	0.96	1.01	0.0035
Location with Recreation	0.45	0.74	0.42	0.73	0.50	0.76	0.0450

Abbreviations: Long-Term Care (LTC); ^a^ Minimum = 0 Maximum = 3; ^b^ Statistically significant findings are in bold.

**Table 3:** Multivariate regression coefficients for CPE selection criteria associated with respondent characteristics.

CPE Selection Criteria	Respondent Characteristics								
	Retail/Community Workplace	≤50 Persons Per mi2	≥14.0% Age 65 or Older	Female Sex	Pharm.D. Degree	Residency	≥10 Years of Experience	BPS Certification ^a^	Preceptor
Licensure Maintenance		−0.18 ^c^						−0.18 ^c^	
Personal Interest									
Self-Improvement			0.13 ^b^	0.25 ^c^					−0.10 ^b^
No/Low Cost				0.13 ^b^			−0.17 ^b^	−0.42 ^b^	
Community/Population Need	0.23 ^c^			0.23 ^c^	0.18 ^b^	0.33 ^c^			−0.18 ^c^
Convenient Location									−0.19 ^b^
Employer Requirement				0.38 ^c^					
Offered Online				0.21 ^c^	0.25 ^c^				
Business Growth	0.42 ^c^	−0.30 ^c^							−0.40 ^c^
Live Credits	−0.22 ^b^				−0.20 ^b^				
Member Organization		−0.20 ^b^						0.28 ^b^	
Location with Recreation									

Abbreviations: Board of Pharmacy Specialties (BPS); Continuing Professional Education (CPE); Doctor of Pharmacy (Pharm.D.); ^a^ Board Certified Ambulatory Care Pharmacist, Board Certified Pharmacotherapy Specialist, Board Certified Psychiatric Pharmacist, and/or; Board Certified Geriatric Pharmacist (formerly Certified Geriatric Pharmacist); ^b^ *p* < 0.05; ^c^ *p* < 0.01.

## References

[1] Allen S.J., Zellmer W.A., Knoer S.J., Phelps P.K., Marvin K.C., Pulvermacher A., Li E., Tyler L.S., Scheckelhoff D.J., Hoffman J.M. (2016). ASHP Foundation Pharmacy Forecast 2017: Strategic Planning Advice for Pharmacy Departments in Hospitals and Health Systems. Am. J. Health Syst. Pharm..

[2] Bryant L.J.M., Coster G., Gamble G.D., McCormick R.N. (2009). General practitioners’ and pharmacists’ perceptions of the role of community pharmacists in delivering clinical services. Res. Soc. Adm. Pharm..

[3] De Grip A. (2006). Evaluating Human Capital Obsolescence.

[4] Doucette W.R., Koch Y.D. (2000). An exploratory study of community pharmacy practice change. J. Am. Pharm. Assoc..

[5] Roberts A.S., Benrimoj S., Chen T.F., Williams K.A., Aslani P. (2006). Implementing cognitive services in community pharmacy: A review of facilitators used in practice change. Int. J. Pharm. Pract..

[6] BeIl J., Standish M. (2005). Communities and health policy: A pathway for change. Health Aff..

[7] Clancy C.M., Cronin K. (2005). Evidence-based decision making: Global evidence, local decisions. Health Aff..

[8] Scott T., Mannion R., Davies H.O., Marshall M.N. (2013). Implementing culture change in health care: Theory and practice. Int. J. Qual. Health Care.

[9] Wilson-Stronks A., Lee K.K., Cordero C.L., Kopp A.L., Galvez E. (2008). One Size Does Not Fit All: Meeting The Health Care Needs of Diverse Populations.

[10] Janke K.K. (2010). Continuing Professional Development: Don’t Miss the Obvious. Am. J. Pharm. Educ..

[11] Lang J.R., Fullerton S.D. (1992). The components of satisfaction with outpatient pharmacy services. Health Mark. Q..

[12] Blodgett J.G., Wakefield K.L., Barnes J.H. (1995). The effects of customer service on consumer complaining behavior. J. Serv. Mark..

[13] The Keys to Staying Competitive in Today’s Healthcare Market. HealthManagement.org. https://healthmanagement.org/c/hospital/whitepaper/the-keys-to-staying-competitive-in-today-s-healthcare-market.

[14] State CE Requirements for Pharmacists; Updated October 2014. http://www.medscape.org/public/pharmcestaterequirements.

[15] Wadelin J.W., Travlos D.V., Janke K.K., Zellmer W.A., Vlasses P.H. (2017). Current and Future Opportunities and Challenges in Continuing Pharmacy Education. Am. J. Pharm. Educ..

[16] United States Census Bureau/American FactFinder (2015). 2011–2015 American Community Survey. U.S. Census Bureau’s American Community Survey Office. http://factfinder2.census.gov.

[17] Marvanova M., Henkel P.J. (2017). Community Pharmacists’ Knowledge of Alzheimer’s Disease Care in High- and Low-Income Chicago. J. Am. Pharm. Assoc..

[18] Marvanova M., Henkel P.J. (2017). Community Pharmacists’ Knowledge Regarding Donepezil Averse Effects and Self-Care Recommendations for Insomnia for Persons with AD. Pharmacy.

[19] APhA-Your Home for Lifelong Learning American Pharmacists Association. https://www.pharmacist.com/education.

[20] Guidance on Continuing Professional Development (CPD) for the Pharmacy Profession Accreditation Council for Pharmacy Education; Updated January 2015. https://www.acpe-accredit.org/pdf/CPDGuidance%20ProfessionPharmacyJan2015.pdf.

